# Effect of a high intake of cheese on cholesterol and metabolic syndrome: results of a randomized trial

**DOI:** 10.3402/fnr.v59.27651

**Published:** 2015-08-19

**Authors:** Rita Nilsen, Arne Torbjørn Høstmark, Anna Haug, Siv Skeie

**Affiliations:** 1 Department of Chemistry, Biotechnology and Food Science, Norwegian University of Life Sciences, Ås, Norway; 2 Section of Preventive Medicine and Epidemiology, Institute of Health and Society, University of Oslo, Oslo, Norway; 3 Department of Animal and Aquacultural Sciences, Norwegian University of Life Sciences, Ås, Norway

**Keywords:** dairy, intervention, Gamalost, Gouda, cardiovascular diseases

## Abstract

**Background:**

Cheese is generally rich in saturated fat, which is associated with increased risk for cardiovascular diseases. Nevertheless, recent reports suggest that cheese may be antiatherogenic.

**Objective:**

The goal of this study was to assess whether intake of two types of Norwegian cheese, with widely varying fat and calcium content, might influence factors of the metabolic syndrome and serum cholesterol levels differently.

**Design:**

A total of 153 participants were randomized to one of three groups: Gamalost^®^, a traditional fat- and salt-free Norwegian cheese (50 g/day), Gouda-type cheese with 27% fat (80 g/day), and a control group with a limited cheese intake. Blood samples, anthropometric measurements, blood pressure, and questionnaires about lifestyle and diet were obtained at inclusion and end.

**Results:**

At baseline, there were no differences between the groups in relevant baseline characteristics, mean age 43, 52.3% female. After 8 weeks’ intervention, there were no changes in any of the metabolic syndrome factors between the intervention groups compared with the control group. There were no increases in total- or LDL cholesterol in the cheese groups compared with the control. Stratified analysis showed that those in the Gouda group with metabolic syndrome at baseline had significant reductions in total cholesterol at the end of the trial compared with control (−0.70 mmol/L, *p=*0.013), and a significantly higher reduction in mean triglycerides. In the Gamalost group, those who had high total cholesterol at baseline had a significant reduction in total cholesterol compared with control (−0.40 mmol/L, *p=*0.035).

**Conclusions:**

In conclusion, cholesterol levels did not increase after high intake of 27% fat Gouda-type cheese over 8 weeks’ intervention, and stratified analysis showed that participants with metabolic syndrome had reduced cholesterol at the end of the trial.

Cardiovascular diseases (CVD) are the most common causes of mortality in the world (1), and lifestyle factors such as dietary changes are successful in reducing the risk of these diseases. Full fat dairy products are rarely recommended in these so-called heart-healthy diets due to the high content of saturated fat in those products, approximately 17% by weight in Norwegian Gouda-type cheeses (2), which is assumed to increase serum cholesterol levels. The Dietary Approaches to Stop Hypertension, for example, recommends a high intake of dairy products, with a focus on predominantly low-fat milk and yoghurt (3). In addition to serum cholesterol, raised serum triglyceride concentrations have long been associated with an increased risk for CVDs; however, whether it promotes CVD or is just a biomarker for risk is still debated (4). Even so, recommendations are to limit the intake of saturated fats, follow a Mediterranean style diet, or to reduce or to maintain serum triglyceride levels below 1.7 mmol/L (4).

On the contrary, observational studies have shown that cheese intake is associated with lower serum triglycerides (5, 6). Furthermore, a higher intake of full fat dairy and total dairy was associated with a better cardiovascular health score than a low intake (7). Intervention trials have also shown that there is some difference within full fat dairy, as cheese intake was shown to lower LDL cholesterol compared with butter intake of equal fat content (8, 9). Cheese and dairy products have also been associated with reduced prevalence (5) and incidence (6) of the metabolic syndrome, a cluster of risk factors for diabetes type 2 and CVD. The findings related to dairy and CVDs are, however, inconsistent and show both a positive effect of cheese intake in women with decreased CVD risk (*p* for trend: 0.03) (10), a negative effect with a 32% higher risk in CVD mortality for each standard deviation increase in high-fat dairy products (11), as well as a favorable cardiovascular risk profile in women, but not in men (12). The reasons for these inconsistencies could be numerous, including different study designs, different outcome measures, different intake levels, and whether they investigate dairy products separately or as a large group encompassing all dairy intakes. Suggested mechanisms of action on the effect of dairy and cheese intake on serum lipids include the effect of bioactive compounds, fatty acids, and micronutrients, specifically calcium (13), as well as inhibition of Δ9-desaturase activity through some unidentified cheese components, possibly related to conjugated linoleic acid (14).

We previously completed a cross-sectional trial to explore whether Gamalost^®^ intake might influence factors of the metabolic syndrome. It was found that intake of Gamalost was negatively associated with systolic blood pressure (BP) (*B*=−0.7, *p=*0.03) (15). Since Gamalost is fat-free, we wanted to investigate experimentally whether intake of either Gamalost or a Gouda-type cheese would influence metabolic syndrome factors. Gouda-type cheeses are the most commonly consumed cheeses in Norway. Unlike most other types of cheese, Gamalost is a traditional Norwegian skimmed milk cheese in that it is naturally free of salt and fat, contains only 160 mg calcium/100 g cheese, and has high protein content and a high amount of bioactive peptides. Details on the production of Gamalost have been previously described elsewhere (16). Norvegia^®^, the Gouda-type cheese included in this trial, contains 27% fat and 800 mg/100 g calcium, making it very different from Gamalost. Since results on dairy intake and factors associated with metabolic syndrome have been inconsistent, and dairy fat content has been implicated, we wanted to compare the effects upon metabolic syndrome factors of these two widely differing cheeses. Possibly, variations in saturated fat, bioactive peptides, and calcium between the cheeses may give different effects on metabolic syndrome factors.

The aim of this trial was accordingly to investigate whether intake of Norvegia or Gamalost cheese might influence factors of the metabolic syndrome, and if they influenced the factors differently.

## Methods

### Subjects

Participants in the trial were recruited from the general population, through local radio, newspapers, and television. We specifically tried to recruit persons with moderately high BP, but normotensive persons were also included. Men and women over 18 years of age who fluently read Norwegian were included. Exclusion criteria were pregnancy and use of BP-lowering medications.

### Design

**Fig. 1:**
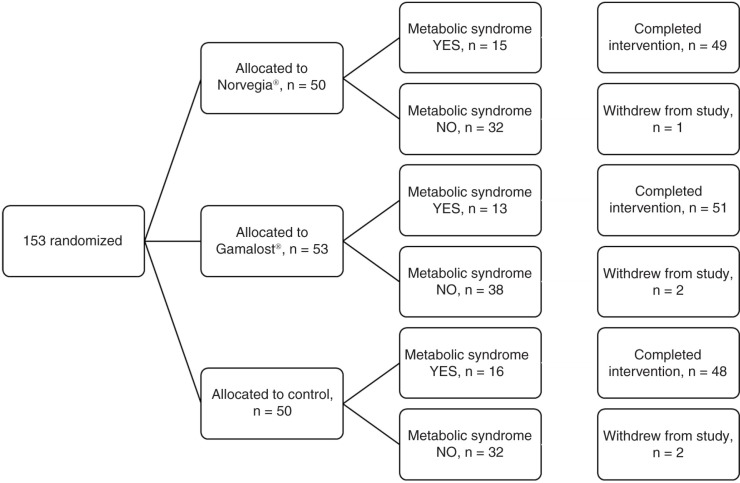
Flow chart of a single-blinded, randomized, controlled trial of Gamalost^®^ and Norvegia^®^ in 153 participants.

This randomized, single-blinded, controlled trial was performed with three parallel arms, which is illustrated in Fig. 1. An 8-week intervention period included measurements taken at baseline and at the end of the trial. The randomization procedure and envelopes containing information as to which arm the participants had been allocated to were prepared by an independent person not involved in the study. Independent of all baseline measurements, the participants were handed the envelopes by two independent persons not involved in the study or the baseline measurements.

This study was carried out at the Department of Chemistry, Biotechnology and Food Science, Norwegian University of Life Sciences, Ås, Norway from April 2013 to July 2013 and was approved by the Regional Committees for Medical and Health Research Ethics (Oslo, Norway) on March 7, 2013 (2013/166) (registered at www.clinicaltrials.gov; NCT01913756). The study was conducted according to the guidelines laid down in the Declaration of Helsinki, and written informed consent was obtained from all subjects.

### Interventions

The participants were randomly assigned to one of three groups – Norvegia, Gamalost, or control. Participants in the cheese groups were asked to maintain their habitual diet, whereas subjects in the control group were asked to limit their intake of the two intervention cheeses. The control group was given a list of cheeses they could consume, consisting mostly of fresh cheeses, blue cheese, and cream cheese. Norvegia and Gamalost are registered trademarks of TINE SA, Norway. The participants consumed 50 g/day or 80 g/day of Gamalost or Norvegia, respectively. These amounts were chosen because they were judged to be higher than the average intake of each cheese, but not so high that the participants were unable to consume the designated amount. Also, to have similar cheese protein intakes in the two cheese groups, the Norvegia intervention cheese amount was larger than the Gamalost intervention. The participants were equipped with digital kitchen scales to accurately weigh out the daily intake. The Gamalost cheeses were all made from the same batch, and they were ripened for 10 days. The Norvegia cheeses were also from the same batch, and they were ripened for approximately 90 days. The participants were provided with all cheese needed for the duration of the study and given more as needed. The nutritional values of the cheeses are presented in Table 1.

**Table 1:** Nutrient composition (per 100 g) of intervention cheeses

Nutrient	Gamalost^®^ (50 g/day)	Norvegia^®^ (80 g/day)
Energy^a^ (kcal)	213 (107)	351 (281)
Protein^a^ (g)	50 (25)	27 (22)
Fat^a^ (g)	1 (0.5)	27 (22)
Saturated (g)	0 (0)	17 (14)
Carbohydrates^a^ (g)	1 (0.5)	0 (0)
Calcium^a^ (mg)	160 (80)	800 (640)
Sodium^a^ (mg)	24 (12)	402 (322)
Magnesium^a^ (mg)	13 (6.5)	33 (26)
Potassium^a^ (mg)	98 (49)	77 (62)

a From TINE SA, manufacturer of Gamalost and Norvegia

Compliance with the cheese intake was judged by evaluation of charts of weighed daily cheese intake, filled out by a subset of the study population.

### Questionnaire

A questionnaire was developed for a cross-sectional trial on Gamalost intake and BP that preceded the current study (15). The questionnaire was a revised version of the previously validated questionnaires used in the cross-sectional Oslo Health Study (the main questionnaire and the second supplementary questionnaire 1 of the Oslo Health Study were used) (17). The baseline questionnaire contained questions about lifestyle, health, medication use, and habitual diet. Some questions focused specifically on dairy product intake. Total dairy product intake was calculated by summarizing the frequency of intake of all cheese, all milk, and fermented milk products. The exclusion questionnaire focused on diet through the trial, difficulty with following the diet, and whether the participants had experienced any discomfort during the intervention. A version of the baseline questionnaire translated into English can be found in Supplementary Material 1.

### Blood samples

Venous blood samples were drawn in the morning between 06:30 and 10:30 after an overnight fast (approximately 10–12 h), using the Vacutainer^®^ system (Becton Dickinson Co., Franklin Lakes, NJ, USA). The samples were centrifuged at 2,500 rpm for 10 min at room temperature, and the serum was separated approximately 1–2 h after the blood was drawn. The serum was frozen to −20°C within 5 h. Fürst Medical Laboratories (Oslo, Norway) conducted the serum analyses. The measured biochemical markers were total cholesterol (mmol/L), HDL cholesterol (mmol/L), LDL cholesterol (mmol/L), and triglycerides (mmol/L). Fasting blood glucose (mmol/L) was measured in capillary blood by the finger stick method, using a LifeScan OneTouch^®^ Verio™Pro (Cilag GmbH International, Schaffhausen, Switzerland).

### Blood pressure

BP was measured using a Microlife^®^ BP A200 sphygmomanometer (Microlife, Widnau, Switzerland). BP was measured after approximately 10 min of rest, in a sitting position and according to the American Heart Association guidelines (18). Three consecutive measurements were taken, and the average of the second and third measurements was recorded. All participants were informed of their BP and whether or not it was within the normal range.

### Anthropometric measurements

Body weight was measured without shoes or heavy clothing, to the nearest 0.1 kg using digital scales (TBF-300A Body Composition Analyzer, Tanita, Tokyo, Japan). Height was measured to the nearest 0.1 cm using a portable stadiometer (Seca 217, Seca, Hamburg, Germany). Body mass index was calculated as weight (kg) divided by the square of height (m). Waist circumference was measured using a measuring tape (Seca 201 Circumference measuring tape, Seca) to the nearest 0.1 cm, according to World Health Organization recommendations, that is, at the midpoint between the iliac crest and the lowest rib margin (19).

### Metabolic syndrome

To qualify as having metabolic syndrome, a person must have at least three of the following five criteria: elevated waist circumference (country-specific cut points,≥94 cm and≥80 cm for European men and women, respectively), elevated triglycerides (≥1.7 mmol/L), reduced HDL cholesterol (<1.0 mmol/L in men and<1.3 mmol/L in women), raised BP (systolic≥130 and/or diastolic≥85 mmHg), or elevated fasting blood glucose (≥5.6 mmol/L) (20). Participants were stratified into two groups for some statistical analyses: MetS-yes if they had metabolic syndrome at baseline, and MetS-no if they did not meet the criteria at baseline. They were also stratified for subgroup analyses by the presence of each individual MetS factor at baseline and categorized as follows: waist-yes/waist-no, TAG-yes/TAG-no, GLU-yes/GLU-no, HDL-yes/HDL-no, SBP-yes/SBP-no, and DBP-yes/DBP-no. Total and LDL cholesterol are not part of the metabolic syndrome and were therefore stratified based on cholesterol guidelines from ‘Adult Treatment Panel III’ (21). Hence, LDL-yes/LDL-no with cut-off at 3.4 mmol/L, and CHOL-yes/CHOL-no with cut-off at 5.2 mmol/L.

### Statistical analysis

Statistical analyses were performed using SPSS 21.0 (IBM Corporation, Armonk, NY, USA). Prior to analyses, the dataset was recoded by an independent person so that the primary researcher was blinded with regard to the intervention group. Data were analyzed according to the intention to treat principle. Baseline characteristics of the study population are presented as mean (standard deviation) or as percentages where appropriate. One-way ANOVA with Bonferroni correction for multiple comparisons or the chi-square (χ^2^) test were used to assess differences between intervention groups at baseline. Paired samples *t*-test was used to assess change in metabolic syndrome factors from inclusion to end of trial in each intervention group.

The Dunnett test was used to evaluate mean changes between each treatment group and the control group. The Dunnett test was also done for the groups stratified by MetS-yes or no, and by each individual factor of the syndrome, as well as total and LDL cholesterol. A *p-*value<0.05 was considered statistically significant.

## Results

### Baseline characteristics

At inclusion, 153 participants were randomized to one of the three groups of the trial (*n=*50 in Norvegiagroup, *n=*53 in Gamalost group, *n=*50 in control group). Five participants were lost to follow-up, one in the Norvegiagroup, and two each in the Gamalost and control groups, as illustrated in Fig. 1. Two participants in the Norvegia group lacked some of the baseline or follow-up measurements due to failure to fast (*n*=1) or failure to complete blood draw (*n*=1), resulting in an effective sample size of 47 for that group. The baseline characteristics of the whole study population and the three groups are presented in Table 2. Approximately, 30% of the population met the criteria to be diagnosed with the metabolic syndrome. As can be seen, there were no major differences in dairy intake or factor of the metabolic syndrome between the three groups at inclusion. LDL cholesterol was higher in the control group than the two other groups (*p=*0.05). As expected from normal Norwegian consumption patterns, the participants had a higher intake of all Gouda-type cheeses (approximately six servings per week) than Gamalost (less than one serving per week).

**Table 2:** Baseline characteristics [mean (SD) or %] for all participants and by intervention groups

	Intervention group								
	
	All (*n*=153)		Norvegia^®^ (*n*=50)		Gamalost^®^ (*n*=53)		Control (*n*=50)		
					
Characteristic	Mean	SD	Mean	SD	Mean	SD	Mean	SD	*p*
Gender, female (%)	52.3		60.0		50.9		46.0		0.4
Age (years)	43.1	16.4	42.7	15.8	41.2	17.0	45.5	16.4	0.4
Weight (kg)	77.2	14.8	76.0	13.6	75.6	13.7	79.9	16.8	0.3
Height (cm)	173.9	8.9	171.9	8.7	174.9	8.7	174.7	9.4	0.2
BMI (kg/m^2^)	25.7	3.7	25.6	3.5	24.6	3.3	26.0	3.7	0.1
Waist circumference (cm)	83.1	11.8	82.8	10.9	80.9	11.3	85.8	12.9	0.1
Systolic BP (mmHg)	132.3	17.2	130.6	14.7	131.5	19.3	134.8	17.2	0.4
Diastolic BP (mmHg)	82.4	9.8	81.4	8.9	82.5	10.6	83.1	10.0	0.7
Total cholesterol (mmol/L)	5.2	1.1	5.3	1.2	5.0	1.2	5.4	1.0	0.2
LDL cholesterol (mmol/L)	2.9	1.0	2.9	1.0	2.7	0.9	3.1	0.9	0.05
HDL cholesterol (mmol/L)	1.7	0.4	1.7	0.4	1.7	0.5	1.6	0.5	0.6
Triglycerides (mmol/L)	1.1	0.6	1.1	0.8	1.0	0.6	1.2	0.5	0.7
Blood glucose (mmol/L)	5.8	0.7	5.7	0.6	5.7	0.9	5.8	0.5	0.7
Metabolic syndrome (%)	30.1		32.0		24.5		34.0		0.5
Education (years)	16.6	2.9	17.0	2.3	16.6	2.7	16.4	3.5	0.6
Smoking^a^ (%)	3.3		2.0		3.8		4.1		0.9
Physical activity^b^ (%)	38.4		36.7		39.6		38.8		0.9
Total dairy^c^	18.4	11.9	17.5	10.1	19.7	12.9	18.0	12.7	0.6
Total cheese^c^	7.5	4.6	7.1	4.2	8.0	4.7	7.2	4.9	0.5
Gouda-type cheeses^c^	5.7	4.3	5.4	3.6	6.1	4.6	5.6	4.8	0.7
Gamalost^c^	0.7	1.9	0.6	1.8	0.6	1.7	0.8	2.2	0.9

SD, standard deviation.

a Percentage daily smokers.

b Percentage who reported moderate to hard physical activity more than 4 h/week.

c Servings per week.

The questionnaire used in this trial was not detailed enough to assess changes in energy or macronutrient intake. However, the questions used to assess food intake showed no changes in food intake, except increased total dairy and cheese intake in the Gamalost and Norvegia groups (data not shown).

All of the individual metabolic syndrome variables were strongly correlated with the whole syndrome (*p*<0.001), and over 90% of participants who met the criteria for the metabolic syndrome had systolic BP over 130 mmHg, 30% had high triglycerides, whereas just 11% of the participants met the low HDL cholesterol criteria (Supplementary Material 2).

### Total and LDL cholesterol changes

As shown in Table 3, a paired samples *t*-test showed that total cholesterol decreased significantly in the entire study population during the intervention, but analyzing the three groups separately, cholesterol was only significantly decreased in the Norvegia group (−0.204 mmol/L, *p=*0.017). Table 4 shows that when stratifying by metabolic syndrome diagnosis, total cholesterol was reduced in MetS-yes participants in the Norvegia group (−0.70 mmol/L, *p*<0.001). Those participants who had high total cholesterol at baseline (Table 5) had significant decreases in total cholesterol in both the Norvegia (−0.39 mmol/L, *p=*0.021) and the Gamalost groups (−0.39 mmol/L, *p=*0.001). Using the Dunnett test to compare changes in the intervention groups with the control group, total cholesterol was only significantly reduced in the Norvegia group (Table 6): for those with MetS-yes, cholesterol was lowered by 0.70 mmol/L (*p=*0.013). Table 6 shows that in those participants who had high total cholesterol at baseline, total cholesterol was decreased in both the Norvegia (−0.39 mmol/L, *p=*0.021) and Gamalost groups (−0.40 mmol/L, *p=*0.035) compared with control.

**Table 3:** Mean difference (MD)^a^ with 95% CI in each group and the whole study population, paired samples *t*-test comparing start to 8 weeks’ follow-up

	Norvegia^®^			Gamalost^®^			Control			Study population		
				
Variable	MD	95% CI	*p*	MD	95% CI	*p*	MD	95% CI	*p*	MD	95% CI	*p*
Total chol (mmol/L)	−0.20	−0.37, −0.04	0.017	−0.09	−0.23, 0.05	0.215	−0.07	−0.25, 0.12	0.477	−0.12	−0.21, −0.03	0.013
LDL chol (mmol/L)	−0.07	−0.21, 0.07	0.342	0.00	−0.12, 0.11	0.959	−0.07	−0.21, 0.07	0.292	−0.05	−0.12, 0.03	0.212
TAG (mmol/L)	−0.06	−0.18, 0.06	0.324	0.02	−0.08, 0.12	0.751	0.13	−0.10, 0.36	0.259	0.03	−0.06, 0.12	0.530
Waist (cm)	−1.0	−1.5, −0.4	0.001	−1.6	−2.1, −1.1	<0.001	−1.0	−1.6, 0.5	0.001	−1.2	−1.5, −0.9	<0.001
SBP (mmHg)	−3.5	−5.8, −1.3	0.003	−4.4	−7.0, −1.8	0.001	−5.4	−8.5, −2.2	0.001	−4.4	−6.0, −2.9	<0.001
DBP (mmHg)	−0.9	−2.6, 0.9	0.319	−2.9	−4.4, −1.4	<0.001	−1.1	−3.3, 1.1	0.333	−1.6	−2.7, −0.6	0.002
Glucose (mmol/L)	0.09	−0.11, 0.28	0.368	0.20	0.00, 0.41	0.051	0.05	−0.16, 0.26	0.634	0.12	0.00, 0.23	0.049
HDL-chol (mmol/L)	−0.04	−0.10, 0.03	0.299	−0.06	−0.10, −0.01	0.010	−0.04	−0.10, 0.01	0.100	−0.05	−0.08, −0.01	0.004

a For baseline values, see Table 2.

**Table 4:** Paired samples *t*-test stratified by positive metabolic syndrome diagnosis (MetS-yes) in each group and the whole study population at baseline^a^

		Norvegia^®^ (*n*=15)			Gamalost^®^ (*n*=13)			Control (*n*=16)			Study population (*n*=44)		
					
Variable		Mean	95% CI	*p*	Mean	95% CI	*p*	Mean	95% CI	*p*	Mean	95% CI	*p*
Total cholesterol	Baseline	6.08			5.25			5.31			5.56		
	Change	−0.59	−0.86, −0.31	<0.001	−0.03	−0.35, 0.29	0.840	0.11	−0.37, 0.58	0.639	−0.17	−0.39, 0.05	0.128
Triglycerides	Baseline	1.77			1.25			1.43			1.49		
	Change	−0.29	−0.56, −0.02	0.039	−0.13	−0.36, 0.11	0.263	0.41	−0.26, 1.07	0.212	0.01	−0.25, 0.28	0.927
Waist circumference	Baseline	90.7			90.5			97.4			93.1		
	change	−1.3	−2.8, 0.1	0.073	−1.9	−3.1, 0.8	0.004	0.1	−1.2, 1.4	0.895	−1.0	−1.7, −0.2	0.011
Systolic BP	Baseline	141.9			150.9			150.1			147.5		
	Change	− 1.8	−7.3, 3.7	0.493	−8.3	−15.9, −0.7	0.035	−10.3	−17.5, −3.1	0.008	−6.8	−10.6, −3.1	0.001
HDL cholesterol	Baseline	1.67			1.58			1.40			1.54		
	Change	−0.03	−0.14, 0.08	0.535	−0.05	−0.13, 0.02	0.148	−0.07	−0.15, 0.02	0.107	−0.05	−0.10, −0.00	0.039

a Only factors with significant associations are shown.

**Table 5:** Paired samples *t*-test stratified by the presence of each metabolic syndrome factor in each group and the whole study population at baseline^a^

		Norvegia^®^			Gamalost^®^			Control			Study population		
					
Variable		Mean	95% CI	*p*	Mean	95% CI	*p*	Mean	95% CI	*p*	Mean	95% CI	*p*
Total cholesterol	*n*	28			20			27			75		
	Baseline	6.04			6.14			6.00			6.17		
	Change	−0.39	−0.62, −0.15	0.002	−0.39	−0.61, −0.17	0.001	0.007	−0.22, 0.23	0.947	−0.25	−0.38, −0.11	0.001
LDL cholesterol	*n*	13			11			16			40		
	Baseline	4.16			3.96			4.01			4.05		
	Change	−0.20	−0.55, 0.15	0.245	−0.32	−0.56, −0.09	0.011	−0.05	−0.28, 0.18	0.655	−0.17	−0.32, −0.02	0.025
Triglycerides	*n*	7			5			6			18		
	Baseline	2.62			2.54			2.08			2.42		
	Change	−0.48	−1.09, 0.13	0.104	−0.64	−0.86, −0.41	0.001	−0.51	−1.33, 0.32	0.175	−0.53	−0.82, −0.24	0.001
Waist circumference	*n*	15			15			18			48		
	Baseline	92.2			94.0			99.2			94.2		
	Change	−0.9	−2.2, 0.5	0.200	−2.4	−3.3, −1.5	<0.001	−0.4	−1.7, 0.9	0.493	−1.2	−1.9, −0.5	0.001
Systolic BP	*n*	25			25			28			78		
	Baseline	141.8			146.8			145.9			144.9		
	Change	−3.5	−6.7, −0.4	0.030	−8.4	−12.4, −4.3	<0.001	−9.0	−13.5, −4.6	<0.001	−7.1	−9.3, −4.8	<0.001
Diastolic BP	*n*	15			19			16			50		
	Baseline	91.7			39.4			94.4			93.2		
	Change	−3.0	−7.3, 1.3	0.156	−5.7	−8.7, −2.7	0.001	−4.1	−7.7, −0.6	0.026	−4.4	−6.3, −2.4	<0.001

a Only factors with significant associations are shown.

**Table 6:** Stratified analysis of significant changes^a^ by MetS (yes or no) or by individual factors of MetS and total cholesterol (yes or no), comparing control group with the two cheese diets

	Baseline mean	End mean	Change	Difference from control (95% CI)	*p*
*MetS-yes*					
Tot CHOL (mmol/L)					
Control	5.45 (0.23)	5.41 (0.29)	0.11 (0.22)		
Norvegia^®^	6.01 (0.33)	5.49 (0.29)	−0.59 (0.13)	−0.70 (−1.25, −0.14)	0.013
Gamalost^®^	5.25 (0.33)	5.22 (0.22)	−0.03 (0.15)	−0.14 (−0.72, 0.44)	0.813
TAG (mmol/L)					
Control	1.46 (0.14)	1.84 (0.30)	0.41 (0.31)		
Norvegia	1.71 (0.27)	1.48 (0.21)	−0.29 (0.13)	−0.70 (−1.38, −0.01)	0.047
Gamalost	1.25 (0.17)	1.12 (0.11)	−0.13 (0.11)	−0.53 (−1.25, 0.18)	0.168
Waist (cm)					
Control	97.6 (2.1)	97.5 (2.2)	0.08 (0.6)		
Norvegia	91.5 (2.6)	89.4 (2.8)	−1.32 (0.68)	−1.40 (−3.35, 0.54)	0.186
Gamalost	90.5 (3.2)	88.6 (3.2)	−1.91 (0.53)	−1.99 (−4.01, 0.03)	0.054
*Individual factors*					
Tot CHOL-yes (mmol/L)					
Control	6.06 (0.14)	6.00 (0.17)	0.01 (0.11)		
Norvegia	6.03 (0.19)	5.66 (0.20)	−0.39 (0.11)	−0.39 (−0.73, −0.05)	0.021
Gamalost	6.11 (0.18)	5.75 (0.14)	−0.39 (0.10)	−0.40 (−0.77, 0.02)	0.035
Waist-yes (cm)					
Control	99.3 (1.75)	98.8 (1.80)	−0.43 (0.62)		
Norvegia	92.3 (2.28)	91.3 (2.35)	−0.85 (0.63)	−0.42 (−2.27, 1.43)	0.828
Gamalost	94.0 (2.11)	91.7 (2.27)	−2.39 (0.43)	−1.95 (−3.80, −0.10)	0.037

Values are mean (SE), two-sided *p*-values for the difference from control (Dunnett test). BP, blood pressure.

a Only factors with significant associations are shown.

LDL cholesterol was reduced in the whole study population for participants who had high LDL at baseline (Table 5) (−0.17 mmol/L, *p=*0.025), but this was only found in the Gamalost group (−0.32 mmol/L, *p=*0.011) when separating the groups. There was no effect on LDL cholesterol when comparing with the control group.

### Metabolic syndrome changes

There were no overall effects of the cheese interventions on the metabolic syndrome as a whole (data not shown), but there were some changes in the individual factors. When stratifying participants by the presence or absence of the metabolic syndrome at baseline, the paired samples *t*-test and the Dunnett test showed some differences in whether or not the participants met the metabolic syndrome criteria. When comparing the change in each metabolic syndrome variable between the intervention groups with the control group, there were no differences between the cheese groups and the control group when analyzing all the participants in each group (data not shown), but again, the stratified analyses showed some changes.

As can be seen from Table 3, paired samples *t*-test showed that waist circumference decreased significantly in the entire study population and in the three groups separately during the intervention. Weight, and thus BMI, was slightly reduced from start to end in the Gamalost and control groups, but, there were no significant changes in either weight or BMI between the groups (data not shown). Table 6 shows that MetS-yes participants in the Gamalost group borderline significantly reduced their waist circumference compared with the control group (−2.0 cm, *p=*0.054). Waist circumference was also significantly decreased in the Gamalost group for those participants with waist-yes compared with the control group (−2.0, *p=*0.037).

As shown in Table 3, there was a slight overall increase in fasting blood glucose in the whole study population (*p=*0.049) which was only borderline significantly present in the Gamalost group when analyzing the three groups separately. There was no significant effect on glucose change when comparing the cheese intervention groups with the control group (data not shown). As can be seen from Table 3, paired samples *t*-test showed that BP decreased significantly in the entire study population during the intervention. All three intervention groups obtained significantly decreased systolic BP during intervention, whereas the Gamalost group was the only group with significant decrease in diastolic BP. There were no differences in systolic or diastolic BP when comparing the cheese groups with the control group at 8 weeks (Nilsen, 2015, unpublished observations). For participants who were MetS-no there were some changes in metabolic syndrome variables, as seen in Supplementary Material 3, but there were no significant differences between the cheese groups and the control group.

Serum triglycerides decreased in the Norvegia group for MetS-yes participants (Table 4) (−0.29 mmol/L, *p=*0.039). As shown in Table 6, compared with the control group those participants who were MetS-yes, significant reductions in triglycerides (−0.70 mmol/L, *p=*0.047) were measured in the Norvegia group. As can be seen from Table 3, there was a slight significant overall decrease in HDL cholesterol (*p=*0.004) in the study population which was only present in the Gamalost group when analyzing the three groups separately. However, this association was lost when comparing the Gamalost group with the control group (results not shown).

## Discussion

The results of this randomized controlled trial suggest a neutral effect on the metabolic syndrome as well as serum cholesterol in participants who consumed a moderate to large amount of the cheeses Norvegia and Gamalost^,^ compared with a control group. When participants were stratified, that is, by MetS at baseline and by each factor of MetS at baseline, the results showed some changes in cholesterol and triglycerides according to cheese intervention group.

### Cholesterol and cheese intake

Total serum cholesterol is not part of the diagnostic criteria for metabolic syndrome; however, it was included in this trial due to its possible relationship with CVDs. The American Heart Association's diet and lifestyle recommendations to prevent CVD make two recommendations related to cheese intake: 1) select fat-free, 1% fat, and low-fat dairy products; and 2) to lower cholesterol, reduce saturated fat to no more than 5–6% of total calories, about 13 g on a 2,000 kcal/day diet (22). In this trial, the participants in the Norvegia group consumed about 14 g/day of saturated fat just from the cheese, but at the end of follow-up there were no increases in total or LDL cholesterol after 8 weeks of increased cheese consumption. Furthermore, those participants in the Norvegia group who were MetS-yes and those who had high cholesterol at baseline had reduced their total cholesterol levels from baseline to the end of the trial, which was also found to be significant when comparing the Norvegia group to the control group of low cheese intake. We are not aware of many similar intervention trials investigating the effect of different cheeses on cholesterol levels, but some results are in accordance with ours and indicate that cheese may not raise cholesterol, as the previously stated recommendations would suggest. Results from an Iranian cross-sectional trial showed that those who consumed cheese more than seven times/week did not have increased cholesterol compared with those who consumed cheese less than seven times/week (23). They also found that lower odds of having metabolic syndrome and low HDL cholesterol if participants had a high cheese intake. However, a cross-sectional trial of adolescents in Portugal found that total cholesterol was significantly higher in the appropriate cheese intake group compared with the low cheese intake group (24). Results from the National Health and Nutrition Examination Survey (NHANES) III show no association between cheese intake and total cholesterol levels in men or women in the United States, but higher frequency of cheese intake was associated with higher HDL cholesterol in women only (12). It is difficult, however, to compare these trials as they are from different countries and habitual diets are likely dissimilar between the three. The three previously mentioned trials were conducted in Iran, Portugal, and the United States, respectively, which are countries with dietary patterns that are distinct from each other. Annual consumption per capita figures show that the average cheese intake in Iran is 4.9 kg 2013; in Portugal it is 9.6 kg (2012), while it is 15.4 kg (2013) in the United States (25, 26). In Norway, the annual consumption was higher than the previously mentioned countries, with 18.1 kg in 2013 (26), or approximately 7.5 servings per week as measured in the current trial.

These contradictory results from cross-sectional studies indicate the need for intervention trials investigating the effect of dairy and cheese on cholesterol under differing habitual diets. Not many intervention trials compare a high cheese intake with a control group of low cheese intake, making it difficult to compare our results with other populations. However, there are some similar trials which show a comparable effect on cholesterol. Total cholesterol was significantly lower on a cheese diet (150 g/8 MJ daily) compared with a diet of butter and casein (27) and a high cheese diet (143 g/day) resulted in 5.7% lower total cholesterol compared with a butter diet (47 g/day) (8). Tholstrup et al. investigated the effect of 205 g hard cheese/10 MJ daily compared with butter and milk intake and found a moderately lower LDL cholesterol after the cheese intervention compared with butter intervention (9). They found no significant effect on total cholesterol which was 0.20 mmol/L higher after the butter intervention compared with cheese intervention (*p=*0.054). It is not completely clear why we have these neutral effects or reductions in cholesterol on a high cheese or high dairy diet. It has been suggested that the main mechanism of action is through calcium, which binds to saturated fatty acids and forms insoluble salts which increase fecal fat excretion, making less saturated fat available for absorption (28). A meta-analysis of randomized controlled trials indicated that increasing calcium intake from dairy by 1,241 mg/day corresponded to an increase of 5.2 g/day of fecal fat excretion (29). A randomized crossover intervention study of 15 healthy men with a 14-day dietary intervention of increased calcium from milk (1,143 mg Ca/10 MJ) or cheese (1,172 mg Ca/10 MJ), or low calcium control group (362 mg Ca/10 MJ) was carried out in Copenhagen from 2011 to 2012 (30). Feces and urine were collected at days 10–14 and 14, respectively, and analyzed for fat and calcium content. Contrary to our results, total and LDL cholesterol increased from baseline in all three groups. However, this effect was attenuated in the cheese and milk groups compared with control. Fecal fat was increased in both milk and cheese groups, and this was correlated with change in both LDL and total cholesterol. In our trial, the Norvegia group consumed 640 mg/day of calcium just from the cheese, indicating that increased calcium intake could be one of the reasons why total cholesterol did not increase even though the participants increased their cheese intake. However, the Gamalost group only consumed 80 mg/day calcium from the cheese, but still had a reduction in total cholesterol compared with control in those participants who had high cholesterol at baseline. This could be a random effect, or it indicates some other mechanism by which cheese may be hypocholesterolemic, for example, possibly related to the presence of bioactive peptides, but further investigations are necessary to support this hypothesis.

The amount of total cholesterol reduction in MetS-yes participants in our trial, about 0.7 mmol/L in the Norvegia compared with control group, may be of clinical significance. A meta-analysis estimated that each 1 mmol/L reduction in total cholesterol corresponded to a 17.5% reduction in relative risk of all-cause mortality (31), hence a reduction of 0.7 mmol/L could contribute to reductions in mortality. Our results show no effect of Norvegia on LDL or HDL cholesterol separately. In the Gamalost group, there was a small but significant decrease in HDL cholesterol compared with baseline in both those who were MetS-no and those who were HDL-no; however, this effect was not present when comparing Gamalost to the control group.

### Metabolic syndrome and cheese intake

Hypertension is a very prevalent condition around the world, and it was estimated that up to 17% of all deaths are attributed to high BP (32). In this trial, over 90% of participants with metabolic syndrome had higher than normal systolic BP, making it the most prevalent criteria of the syndrome. However, after 8 weeks of intervention, there were no significant effects of the cheeses on BP in this trial. Several studies have shown positive effects of dairy product intake on MetS or single factors of the MetS. However, there are inconsistencies and variations in study design and in which dairy products are studied. In the observational Oslo Health Study, results showed that the frequency of cheese intake was significantly negatively associated with serum triglycerides, diastolic BP, and waist circumference, and positively associated with HDL cholesterol (5). Similarly, a French prospective observational study found that frequency of cheese intake was negatively associated with triglyceride levels and also lower increase in waist circumference over 9 years (6). Another French prospective observational trial found no effect of cheese intake on factors of MetS in men or women; however, when stratifying by baseline BMI, cheese intake was significantly positively associated with HDL cholesterol and negatively with fasting glucose in those who had a BMI<25 kg/m^2^ (33). These trials did not differentiate between different types of cheese, meaning the results could be associated with any kind of cheese. Only about one third of the MetS-yes participants in our trial had high triglycerides, making it one of the least prevalent abnormalities. It has been known for a long time that serum triglycerides is associated with CVD risk, independent of other risk factors (34), but the effect of dietary change on triglycerides is less well established. A meta-analysis of randomized controlled trials found that low-fat diets had no effect on serum triglycerides in women (35) and neither did low glycemic index diets (36).

There are no good figures for the prevalence of metabolic syndrome in the general healthy Norwegian population. The International Diabetes Federation estimates that about 25% of the world's population have metabolic syndrome (37), whereas the prevalence in healthy non-diabetic Europeans was approximately 15% in 2004 (38). In the current trial, about one third of the population met the criteria, indicating that the prevalence has either increased over the past 10 years, or that our population is not representative of the general European population. However, the different metabolic syndrome definitions used can also influence the prevalence, as the European trial used a modified WHO definition where hyperinsulinemia had to be present to be diagnosed. Furthermore, we specifically tried to recruit participants with moderately high BP, which obviously has an effect on the prevalence of metabolic syndrome in this study.

The design of the study itself is the main strength of this trial. The duration of the intervention was quite long and the population fairly large compared with similar trials, and the randomization allowed for three groups of similar characteristics at baseline. The Norvegia intervention cheese is the most commonly consumed cheese in Norway, making the results relevant to a large part of the population. A limitation of this trial is the possibility of the results being influenced by the statistical phenomenon known as regression to the mean (39). If the baseline measurements were falsely high, it is possible that reductions would occur on subsequent visits, irrespective of the interventions. However, since the subjects were randomly allocated to the groups, it can be argued that the regression to the mean is equal in all groups, and therefore that the results represent a true change.

In conclusion, even though cheese and high-fat dairy products are not recommended in heart-healthy diets, results from this trial do not show a negative effect of cheese intake on cholesterol or metabolic syndrome. Consuming 80 g/day of Norvegia, a 27% fat Gouda-type cheese, appeared to have a slight hypocholesterolemic effect in those participants who had metabolic syndrome and high cholesterol at baseline, compared with the control group of low cheese intake. Additional studies are needed to confirm these results, as well as to investigate the effect of other cheeses.

## Supplementary Material

Effect of a high intake of cheese on cholesterol and metabolic syndrome: results of a randomized trial
